# The Impact of Multiple Biologic Failures on Surgical Outcomes: A Retrospective Cohort Study of Elective Subtotal Colectomy for Ulcerative Colitis

**DOI:** 10.3390/jcm15208123

**Published:** 2026-10-19

**Authors:** Yaron Rudnicki, Jaxson R. Jeffery, Donya Derakshani, Kellie L. Mathis

**Affiliations:** 1 Department of Surgery, Division of Colon and Rectal Surgery, Mayo Clinic, Rochester, MN 55905, USA; 2 Department of Surgery, Meir Medical Center, Gray School of Medicine, Gray Faculty of Medical and Health Sciences, Tel Aviv University, Tel Aviv 6997801, Israel

**Keywords:** ulcerative colitis, medical refractory disease, subtotal colectomy, biologic agents

## Abstract

**Background:** Subtotal colectomy (STC) with end ileostomy (EI) is usually the first surgical stage for patients with ulcerative colitis (UC) who fail medical treatment, most often with biological agents. Is there a difference in surgical outcome after failure of one or two biologicals vs. three or four? **Methods:** A retrospective study of all patients who underwent STC with EI for UC (5-2018 to 1-2022). After excluding emergent cases, the elective cohort was divided into patients who failed 1–2 biologicals (group 1–2) vs. patients who failed 3–4 biologicals (group 3–4), and surgical outcomes were compared. Data collected included patients’ demographics, preoperative clinical manifestations, and operative and postoperative outcomes. **Results:** Ninety-eight patients underwent STC for UC, and of them, 53 were elective. Thirty patients failed 1–2 biologics (group 1–2), and 23 failed 3–4 biologics (group 3–4). The groups were similar in average age (37.6 ± 11 vs. 32.7 ± 10), BMI (26.7 ± 6 vs. 27.7 ± 7), number of years with UC (9.5 ± 9 vs. 9 ± 8), rate of steroid dependence (20 (67%) vs. 13 (57%)) but differed in sex; there were 25 (83%) males in group 1–2, and 8 (35%) in group 3–4 (*p* < 0.01). The operative approaches were similar, as was the operative time (217 ± 74 vs. 210 ± 79 min). The length of hospital stay was 3.2 ± 1 days for group 1–2 and 5 ± 6 days for group 3–4 (*p* = 0.14). Postoperative complications were seen in 10 patients (33%) vs. 7 patients (30%) (*p* = 0.82), and of them, two (7%) and two (9%) (*p* = 0.82) had Venous thromboembolism (VTE), respectively. Only 20 patients (67%) in group 1–2 and 16 patients (70%) in group 3–4 (*p* = 0.82) progressed to a second stage of an Ileoanal pouch (IPAA) at 11.1 ± 4 and 9.2 ± 2 months, respectively. The overall average follow-up time was 41.1 months. **Conclusions:** Ulcerative Colitis patients who fail 3–4 biological agents and choose to go to elective STC with EI have very similar surgical outcomes compared to patients who choose elective surgery after failing only 1–2 biological agents in the same time span of known disease. UC patients with severe colitis and poor quality of life who fail two lines of biological agents may be better off electing surgical treatment than failing more biologics and then going to surgery. On the other hand, patients who try 3–4 biologics may expect no worse surgical outcomes than after 1–2 biologics.

## 1. Introduction

The prevalence of Ulcerative Colitis (UC) among the American population is around 260 patients per 100,000 people and is rising [1,2]. Although medical treatment has improved the lives of UC patients immensely, some patients will fail medical treatment and will undergo surgery at some point in their lives. Surgery is indicated for various reasons, ranging from severe acute colitis to colorectal malignancy, but most often due to intractability and failure to respond to medical treatment [3,4]. Intractability is characterized by poor quality of life, an inability to control symptoms, difficulty adhering to medical therapy, or failure to thrive.

When opting for the surgical approach, a subtotal colectomy (STC) with end ileostomy (EI) is usually the first surgical stage for patients with UC, and it is the procedure of choice. This approach allows patients to recuperate after the removal of most of the diseased colon, regain weight, control most of their symptoms, improve their quality of life, reduce the need for medications, and open the door for a future restorative operation [5].

Most recent guidelines for treating and managing UC recommend biological agents as first-line treatment for moderate to severe disease and second-line for mild-to-moderate disease [6]. The percentage of UC patients treated with biologicals in 2017 in the US was 16.2% and is persistently rising [7]. The introduction of biological agents has reduced the rates of emergency surgery dramatically in the setting of acute severe UC, allowing most of these patients to avoid surgery in the acute phase [8]. On the other hand, the rate of overall UC patients who undergo surgery has not changed in the last two decades and has stayed stable at 10% [9]. A subset of UC patients will need surgery at a certain point in their lives, no matter how much medical treatment they receive, with most surgeries performed in an elective setting [10]. Identifying the right patients who will benefit from elective surgery is always challenging. On one hand, there is added morbidity with such extensive surgery, but on the other hand, most of these patients endure a very poor quality of life, which at times is prolonged with unsuccessful attempts with more biological agents. While previous studies have evaluated general elective surgical outcomes in the biologic era, this study aims to specifically quantify the comparative surgical risk for UC patients undergoing elective STC with EI after the sequential burden of failing 1–2 vs. 3–4 biological treatments.

## 2. Patients and Methods

A retrospective study was designed to identify all patients who underwent a subtotal colectomy (STC) with an end ileostomy (EI) for UC at Mayo Clinic from May 2018 to January 2022, out of a prospectively maintained institutional database. The study was conducted in accordance with the ethical principles of the Declaration of Helsinki (Edinburgh 2000) and the approval of the Institutional Review Board. Patients with a diagnosis of UC who were treated with at least one biologic agent and elected to undergo surgery for removal of most of their colon (STC) were included in the study. Patients who were initially hospitalized for severe colitis, failed conservative treatment, and underwent an emergent STC were excluded from the study to allow for a better assessment of the compound impact of multiple biological agents.

The remaining cohort of UC patients who underwent an elective STC was divided into two groups according to the number of biological agents that they were treated with and failed before electing for surgery. Treatment failure of a biological agent was clinically defined as the inability to achieve or maintain symptom remission, necessitating either a switch to a different biologic agent or the decision to proceed with surgical intervention. Patients who failed one or two biological agents (Group 1–2) were compared with those who failed three or four (Group 3–4). There were no patients who received more than four biologicals in the cohort. The basis for the separation between Group 1–2 and Group 3–4 was clinical practice, where the first one or two biologic agents often represent the standard initial therapeutic strategies according to the American Gastroenterological Association (AGA) guidelines [11]. On the other hand, the failure of a second biologic agent is a significant clinical milestone and often indicates a higher degree of disease severity or a more complex inflammatory mechanism that is resistant to standard approaches [12].

The primary outcomes of the study were the rate of postoperative complications and the length of hospital stay as main markers of postoperative outcome. The secondary outcome was the rate of venous thromboembolism (VTE), as patients undergoing STC are at a higher risk. Data collected included demographic information such as age, sex, preoperative clinical manifestations, laboratory markers, medications used, operative findings, and approaches. Postoperative outcomes were measured by length of stay, postoperative complications, rate of venous thromboembolism (VTE) events, and rates of patients who continued to the second stage of reconstructive surgery. Postoperative complications were defined as adverse clinical events occurring following surgery, specifically including high-output ileostomy, prolonged ileus, small bowel obstruction, surgical site infection, sepsis, hemorrhage requiring transfusion, venous thromboembolism (VTE), and rectal stump leak. To standardize the assessment of preoperative disease severity, clinical activity was retrospectively categorized for each patient using the Truelove and Witts severity index, based on their bowel movement frequency, presence of visible blood in the stool, temperature, hemoglobin levels, and C-reactive protein (CRP) levels.

Descriptive statistics were used to summarize patient demographics and clinical characteristics. Continuous variables are presented as means and standard deviations (SD) and were compared between the two groups using the independent samples Student’s *t*-test or the Mann–Whitney U test, as appropriate for data distribution. Categorical variables are presented as frequencies and percentages and were compared using Pearson’s Chi-square test, or Fisher’s exact test when expected cell counts were small. A two-sided *p*-value of <0.05 was considered statistically significant. All statistical analyses were performed using SPSS version 28.0.

## 3. Results

A total of 98 patients with UC underwent a subtotal colectomy (STC) with an end ileostomy (EI) during the study period. Of them, 53 patients had surgery as an elective procedure and were the focus of this study. The mean age of patients electing to go for an STC was 35 ± 11 years, 55% male, with an average body mass index (BMI) of 27.1 ± 6.4 kg/m2 and a diagnosis of UC for an average of 9.3 ± 8.6 years. All UC patients were previously treated with at least one biological agent prior to surgery. The most common biologics used were anti-TNF-α agents such as Infliximab (83%), Adalimumab (55%), and Golimumab (8%), IL-12 and IL-23 agents such as Ustekinumab (23%), anti-integrin agents such as Vedolizumab (79%), and JAK inhibitors like Tofacitinib (34%). In addition to biological agents, 98% were previously treated with 5-aminosalicylic acid (5-ASA), 15% with Sulfasalazine, and 13% with Methotrexate; 62% of patients received at least one course of corticosteroids in the three months up to surgery, and of them, 50% were considered steroid-dependent and went in for surgery on steroids. None of the patients were on intravenous steroids before surgery. The average number of different medical therapies (biological and non-biological) that patients had used and failed before surgery was 4.4 ± 1.5 (range of 2–7); 10% of patients had a previous infection with colonic Cytomegalovirus (CMV), and 30% had a history of *Clostridioides difficile* (C. diff.) *colitis*.

The elective cohort was divided into two groups according to the number of biological agents they failed on before electing to go to surgery. Thirty patients who were treated with only one or two biologicals (Group 1–2) were compared to 23 patients who failed more than two, mainly three or four biologicals (Group 3–4). There were 25 (83%) males in group 1–2, and only 8 (35%) in group 3–4, *p* < 0.01. The groups were similar in all other regards: age (37.6 ± 11 vs. 32.7 ± 10 years, *p* = 0.1), body mass index (BMI) (26.7 ± 6 vs. 27.7 ± 7 kg/m^2^, *p* = 0.63), and the average number of years with UC (9.5 ± 9 vs. 9 ± 8, *p* = 0.81), respectively. The groups were also similar in pre-operative clinical presentations, with group 1–2 and group 3–4 had similar rates of patients with pre-operative flare-up (30% vs. 22%, *p* = 0.49), similar mean duration of the flare (106 ± 69 vs. 126 ± 39 days, *p* = 0.8), average number of bowel movements (BM) per day (9.2 ± 7 vs. 9.3 ± 6, *p* = 0.95), percentage of patients with bloody BM (60% vs. 78%, *p* = 0.16), chronic abdominal pain (60% vs. 82%, *p* = 0.08), Severe urgency (40% vs. 48%, *p* = 0.57) and the rate of patients that lost more than ten Lbs. in the year up to surgery (27% vs. 30%, *p* = 0.69), respectively. The groups also had similar pre-operative laboratory presentations regarding inflammatory markers such as CRP, WBC, Hemoglobin levels, and albumin levels; 67% of Group 1–2, and 57% of Group 3–4 were steroid dependent up to surgery (*p* = 0.45), the average overall number of immunomodulators medications failed on were 3.5 ± 1 for Group 1–2 and 5.6 ± 1 for Group 3–4 (*p* < 0.01) and the average biological agents were 1.6 ± 0 for Group 1–2 and 3.3 ± 0 for Group 3–4 (*p* < 0.01) (Table 1). Utilizing the Truelove and Witts criteria, 18 patients (60%) in Group 1–2 and 18 patients (78%) in Group 3–4 were classified as having severe clinical disease activity prior to elective surgery (*p* = 0.16).

### Surgical Approach

Twenty percent of Group 1–2 and 13% of Group 3–4 were characterized by an American Society of Anesthesiologists (ASA) score of three or above. All patients went to surgery for a subtotal colectomy with an end-Ileostomy. Laparoscopic subtotal colectomy with end Ileostomy was performed in 19 (63%) patients in Group 1–2 and 14 (61%) in Group 3–4, Hand assisted Laparoscopic subtotal colectomy with end Ileostomy in 10 (33%) and 6 (26%), Robotic subtotal colectomy with end Ileostomy in 1 (3%) and 2 (9%), respectively, and one patient in Group 3–4 had an open subtotal colectomy with end Ileostomy. The rectal stumps were only stapled in the pelvis in 12 (40%) and 15 (65%) patients (*p* = 0.07), while 18 (60%) and 8 (35%) had the stapled line oversewn (*p* = 0.07), and the operative time was 217 ± 74 vs. 210 ± 79 min, respectively (Table 2).

**Figure 1:**
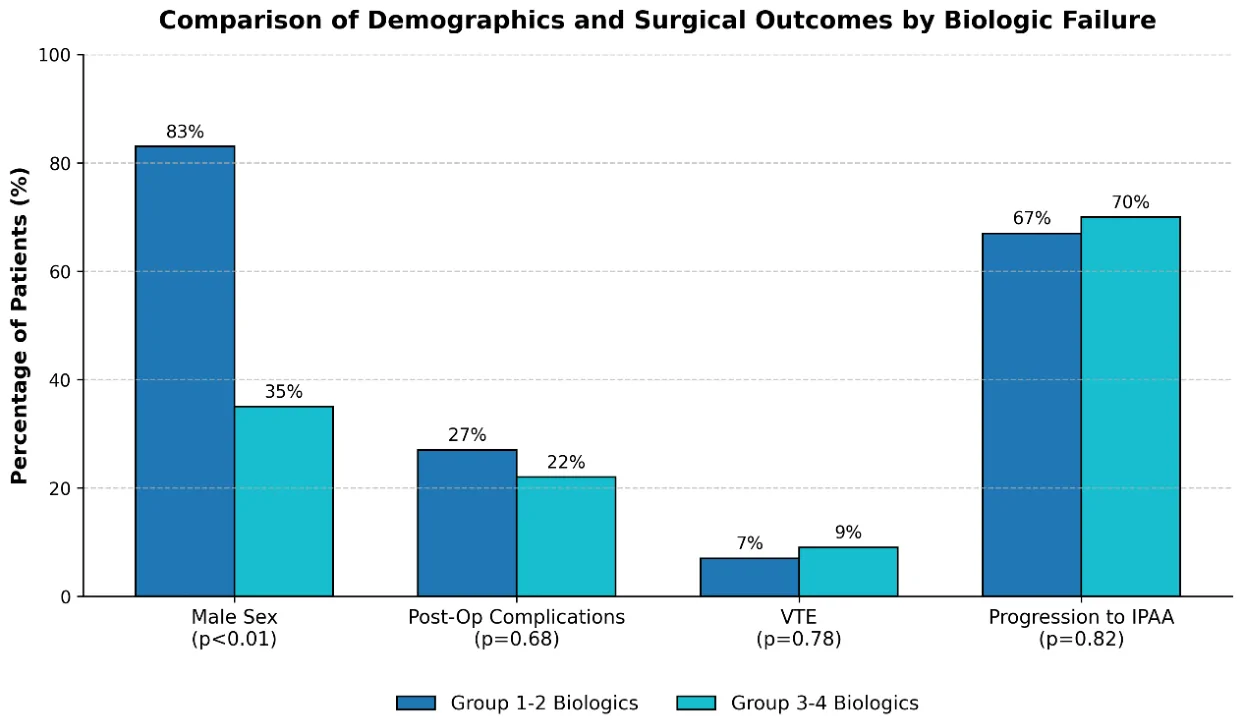
Sex distribution and primary surgical outcomes for patients failing 1–2 versus 3–4 biological agents prior to elective subtotal colectomy. Despite a significant baseline sex difference (*p* < 0.01), rates of complications, VTE, and progression to IPAA remain statistically equivalent.

The length of hospital stay (LOS) differed, with 3.2 ± 1 days for group 1–2 and 5 ± 6 days for group 3–4 (*p* = 0.14). Overall, eight (27%) patients in Group 1–2 and five (22%) in Group 3–4 had postoperative complications (*p* = 0.68); a few of them had more than one. High-output ileostomy was seen in five and two patients, and Ileus in four and three, respectively. Group 3–4 had one case of anemia needing RBC transfusion, two cases of small bowel obstruction, and one case of SSI and sepsis. The rate of post-operative venous thromboembolism (VTE) was two (7%) and two (9%) patients (*p* = 0.82), with portal vein thrombosis seen in two patients in Group 3–4 and in one in Group 1–2, and one case of Pulmonary embolism (PE) in Group 1–2. There were no cases of deep vein thrombosis (DVT) and no cases of rectal stump leak (Table 3). Only 20 patients (67%) in Group 1–2 and 16 patients (70%) in Group 3–4 (*p* = 0.82) progressed to a second stage of an Ileoanal pouch (IPAA) at 11.1 ± 4 and 9.2 ± 2 months, respectively. The overall average follow-up time was 37.7 ± 28 and 44.9 ± 25 (*p* = 0.34), respectively (Figure 1).

## 4. Discussion

The management of medically refractory ulcerative colitis (UC) is a clinical crossroads where the promise of novel medical therapies must be weighed against the definitive resolution offered by surgery. This study sought to clarify the surgical risks associated with prolonged medical management, specifically questioning whether the cumulative burden of failing multiple biologic agents translates into worse outcomes following elective subtotal colectomy (STC). A subtotal colectomy with an ileostomy is the procedure of choice for these patients. Removing the bulk of the disease allows the patient’s health to be restored, medication to be withdrawn, and permits a future restorative operation [5].

There are no universal guidelines that dictate the optimal time to transition from medical to surgical treatment based on a specific number of failed biologic agents. Instead, the decision is guided by a set of clinical principles and indications that focus on the patient’s overall condition, quality of life, and failure to meet treatment goals [13]. On the other hand, there are no guiding principles to decide when the biological treatment has run its course, and what is the number of agents a patient needs to fail on to realize that additional medical treatment is futile. The most common reason for elective surgery in these patients is medically refractory disease, also known as intractability. This is broadly defined by persistent symptoms, poor quality of life, and an inability to control the disease despite maximal medical therapy [3]. The goal of medical treatment is to achieve and maintain a sustained, steroid-free remission; failure to reach this goal should be a primary trigger for considering surgery [14].

This study suggests that in the elective setting, patients who have failed three or four biologic agents experience postoperative outcomes that appear comparable to those who have failed only one or two, though these findings must be interpreted with caution given the cohort size. The core implication of this study is that for patients with intractable UC, prolonging a course of ineffective medical therapy offers no discernible advantage in mitigating surgical risk. Despite the multi-failure group representing a more refractory disease phenotype—evidenced by a significantly higher number of failed immunomodulator medications—there were no significant differences in primary surgical outcomes. Rates of overall postoperative complications (33% vs. 30%), venous thromboembolism (7% vs. 9%), and progression to a second-stage ileal pouch-anal anastomosis (IPAA) (67% vs. 70%) were nearly identical. By deliberately excluding emergent cases, this analysis highlights the crucial window provided by the elective pathway for patient optimization. Corticosteroids can be weaned, nutritional deficits corrected, and acute inflammation managed. This process may act as an equalizer, mitigating the inherent risks associated with a more severe disease history, bringing patients to a similar baseline of surgical fitness.

Perhaps the most compelling result of this study is the profound sex difference between the cohorts. The group proceeding to surgery after fewer biologic failures was overwhelmingly male (83%), whereas the group that endured a longer medical course was predominantly female (65%). This suggests that male and female patients may follow fundamentally different pathways to the operating room, showing a striking sex disparity in the path to surgery. While some large database studies have found no significant sex-based differences in rates of biologic use or surgical intervention [15]. Others have reported that men are more likely to undergo definitive surgery while women are more likely to be treated with short-term rescue therapies [16]. The drivers are likely multifactorial. For female patients of childbearing age, valid concerns regarding the impact of pelvic surgery on future fertility and sexual function are paramount. The psychosocial impact of an ileostomy on body image may also contribute to a greater willingness to trial additional medical therapies. While this study cannot elucidate the cause, highlighting this disparity so starkly underscores the need to incorporate sex- and gender-specific analyses into future IBD research and clinical counseling.

The decision to change course from trying another biological agent after one or two agents have failed and choosing elective major surgery is not an easy one to make. Most often, it is the treating gastroenterologist who is leading the conversation on what the next step is for that patient. This study affirms that the equivalence of surgical risk, irrespective of the length of the preceding medical journey, provides a strong rationale for reframing the conversation around the timing of surgery, advocating for its consideration as a timely and effective intervention rather than a last resort. For a patient suffering from a poor quality of life, debilitating symptoms, and steroid dependency after failing two lines of biologic therapy, the pursuit of a third or fourth agent may only prolong a period of significant distress.

Studies investigating patient perspectives have found that a substantial number of individuals who underwent surgery for UC wish they had done so sooner (44–53%), citing the prolonged suffering from intensive and ultimately unsuccessful drug treatments as a primary reason [13,17,18]. Our findings empower clinicians to shift the conversation from “let’s try one more drug” to “surgery is a safe and effective option now, with outcomes no better than if we wait.” This reframes surgery not as a failure of medical management, but as a proactive and definitive step toward restoring health and quality of life. These findings should empower gastroenterologists and surgeons to have earlier, data-driven conversations about operative intervention. 

The primary limitations of this study revolve around its retrospective, single-center design and modest sample size. Comparing 30 patients to 23 patients inherently carries a risk of a Type II error, meaning the study may be underpowered to detect true, more subtle differences in postoperative morbidity or complication rates between the cohorts. The small sample size precluded a robust multivariate regression to adjust for sex. Furthermore, the lack of granular data on patient-reported outcomes and quality-of-life metrics prevents a full quantification of the burden of delaying surgery. Additionally, because many patients had their initial endoscopic evaluations at outside institutions, we lacked complete data to accurately report the baseline Montreal classification for disease extent. As a single-center study, our cohort may represent a more complex patient population, making these findings most generalizable to other specialized IBD centers rather than community practices.

## 5. Conclusions

In patients undergoing elective subtotal colectomy for medically refractory ulcerative colitis, the number of previously failed biologic agents does not appear to be associated with an increased risk of postoperative morbidity. Surgical outcomes were equivalent for patients who failed one or two biologics compared to those who failed three or four. While these findings do not inherently mandate earlier surgical intervention, they suggest that trialing additional lines of biologic therapy does not worsen the baseline surgical risk profile once intractability is established. Therefore, elective surgery remains a safe and equally viable option at any point along this medical treatment continuum.

## Figures and Tables

**Table 1:** Baseline Demographics, pre-operative clinical and laboratory presentations.

Characteristics	Group 1–2 Biologicals *n* = 30	Group 3–4 Biologicals *n* = 23	*p*-Value
Male—*n* (%)	25 (83%)	8 (35%)	<0.01
Age at Op—mean ± SD	37.6 ± 11	32.7 ± 10	0.10
BMI—mean ± SD	26.7 ± 6	27.7 ± 7	0.63
Number of years with UC—mean ± SD	9.5 ± 9	9 ± 8	0.81
** Pre-Op Clinical presentation:**			
Pts with pre-Op flare-up—*n* (%)	9 (30%)	5 (22%)	0.49
Duration of pre-Op flare-up (days)—mean ± SD	106 ± 69	126 ± 39	0.80
Bowel movements per day—mean ± SD	9.2 ± 7	9.3 ± 6	0.95
Pts with Bloody bowel movements—*n* (%)	18 (60%)	18 (78%)	0.16
Pts with Abdominal pain—*n* (%)	18 (60%)	19 (82%)	0.08
Severe Urgency—*n* (%)	12 (40%)	11 (48%)	0.57
Pts with >10 Lb. Lost up to OP—*n* (%)	8 (27%)	7 (30%)	0.69
Pts with Fever—*n* (%)	1 (3%)	0	0.38
** Pre-Op Laboratory findings:**			
CRP—mean ± SD	14.9 ± 21	14.6 ± 13	0.95
WBC—mean ± SD	9.5 ± 3	10.3 ± 4	0.46
Hemoglobin–mean ± SD	12.8 ± 2	13.1 ± 2	0.59
Albumin—mean ± SD	4 ± 2	4.1 ± 2	0.39
History of CMV—*n* (%)	3 (10%)	2 (9%)	0.87
History C-Diff—*n* (%)	10 (33%)	6 (26%)	0.59
** Pre-Op Medication:**			
Steroid Dependent—*n* (%)	20 (67%)	13 (57%)	0.45
Number of overall medications failed on—mean ± SD	3.5 ± 1	5.6 ± 1	<0.01
Number of Biologics failed on—mean ± SD	1.6 ± 0	3.3 ± 0	<0.01
Infliximab—*n* (%)	25 (83%)	19 (83%)	<0.01
Vedolizumab—*n* (%)	17 (57%)	23 (85%)	
Adalimumab—*n* (%)	6 (20%)	21 (78%)	
Tofacitinib—*n* (%)	7 (23%)	9 (33%)	
Ustekinumab—*n* (%)	1 (3%)	9 (33%)	
Golimumab—*n* (%)	0	4 (15%)	

**Table 2:** Operative data and approach.

Characteristics	Group 1–2 Biologicals *n* = 30	Group 3–4 Biologicals *n* = 23	*p*-Value
ASA Score ≥ 3—*n* (%)	6 (20%)	3 (13%)	0.50
**Type of Op**			
Laparoscopic Subtotal colectomy with end Ileostomy—*n* (%)	19 (63%)	14 (61%)	0.85
Hand-assisted Laparoscopic Subtotal colectomy with end Ileostomy—*n* (%)	10 (33%)	6 (26%)	0.57
Robotic Subtotal colectomy with end Ileostomy—*n* (%)	1 (3%)	2 (9%)	0.40
Open Subtotal colectomy with end Ileostomy—*n* (%)	0	1 (4%)	0.25
**Rectal stump treatment**			
Stapled in the pelvis—*n* (%)	12 (40%)	15 (65%)	0.07
Oversewn stapled line—*n* (%)	18 (60%)	8 (35%)	0.07
Op duration (min)—mean ± SD	217 ± 74	210 ± 79	0.89

**Table 3:** Post-operative outcome and complications.

Characteristics	Group 1–2 Biologicals *n* = 30	Group 3–4 Biologicals *n* = 23	*p*-Value
Length of stay (days)—mean ± SD	3.2 ± 1	5 ± 6	0.14
Post Op complications—*n* (%)	8 (27%)	5 (22%)	0.68
Anemia–Transfused RBC	0	1	
High-output ileostomy	5	2	
Ileus	4	3	
Small bowel obstruction	0	2	
Sepsis	0	1	
Venous thromboembolism (VTE)—*n* (%)	2 (7%)	2 (9%)	0.78
Pulmonary embolism (PE)—*n* (%)	1	0	
Portal VTE—*n* (%)	1	2	
DVT—*n* (%)	0	0	
Went for the 2nd stage IPAA	20 (67%)	16 (70%)	0.82
Time interval from 1st to 2nd stage (months)	11.1 ± 4	9.2 ± 2	0.09
Follow-up (Months)	37.7 ± 28	44.9 ± 25	0.34

## Data Availability

The original contributions presented in this study are included in the article. Further inquiries can be directed to the corresponding author.
